# Efficacy and safety of single high-dose intravenous ferric carboxymaltose in patients undergoing hemodialysis: a multicenter, single-arm, open-label, clinical trial

**DOI:** 10.1186/s12882-026-05153-z

**Published:** 2026-06-29

**Authors:** Kosuke Osawa, Shin-ichi Araki, Yusuke Tanaka, Masaki Ohya, Toshihiro Kodama, Yasushi Saika, Shigeo Negi, Shuto Yamamoto, Tsunehisa Matsuo, Yoji Ogawa, Norifumi Katayama, Yusuke Shigi, Tomomi Naito, Toshio Shimokawa, Takashi Shigematsu

**Affiliations:** 1 Department of Nephrology, School of Medicine, Wakayama Medical University, 811-1 Kimiidera, Wakayama, Wakayama, 641-8509 Japan; 2 Department of Nephrology, Kindai University Nara Hospital, 1248-1 Otoda-Cho, Ikoma, Nara, 630-0293 Japan; 3 Department of Internal Medicine, Kinokawa Clinic, 501-1 Nishikokubu, Iwade, Wakayama, 649-6213 Japan; 4 Department of Nephrology, Fujii Hospital, 3-1 Nishinouchicho, Kishiwada, Osaka 596-0044 Japan; 5 Department of Internal Medicine, Taniguchi Hospital, 327-11 Hikata, Kainan, Wakayama, 642-0002 Japan; 6 Department of Internal Medicine, Habara Hospital, 1-1-4 Hagurazaki, Izumisano, Osaka 598-0046 Japan; 7 Department of Internal Medicine, Wakaura Central Hospital, 6-2-70 Shioya, Wakayama, Wakayama, 641-0054 Japan; 8 Department of Internal Medicine, Nishioka Hospital, 278-1 Kojima, Aridagawa, Wakayama, 643-0034 Japan; 9 Department of Nephrology, Naga Municipal Hospital, 1282 Uchita, Kinokawa, Wakayama, 649-6414 Japan; 10 Department of Biostatistics, School of Medicine, Wakayama Medical University, 811-1 Kimiidera, Wakayama, Wakayama, 641-8509 Japan; 11 Division of Nephrology, Rinku General Medical Center, 1-1-1 Rinku Ourai-Kita, Izumisano, Osaka 598-8577 Japan

**Keywords:** Ferric carboxymaltose, Hemodialysis, Anemia, Iron

## Abstract

**Background:**

Iron-deficiency anemia is common in patients undergoing hemodialysis, and intravenous iron therapy is often preferred because oral iron is poorly tolerated and less effective. Ferric caboxymaltose, a high-dose intravenous iron formulation, has recently become available in Japan. However, evidence regarding its efficacy and safety in hemodialysis patients remains limited. Therefore, this study investigated the efficacy and safety of a single intravenous administration of ferric carboxymaltose containing 500 mg of iron in Japanese patients undergoing hemodialysis.

**Methods:**

A 12-week, multicenter, prospective, open-label, single-arm trial was conducted, enrolling 30 Japanese patients undergoing hemodialysis with hemoglobin < 10 g/dL and iron-deficiency (serum ferritin < 100 ng/mL or transferrin saturation [TSAT] < 20%). Hemoglobin, iron-related parameters (serum ferritin, TSAT, 8-hydroxydeoxyguanosine [8-OHdG], hepcidin, intact fibroblast growth factor 23 [FGF-23], and phosphate), and quality of life (Short Form-36 Health Survey [SF-36] and EuroQol 5-Dimension 5-Level) were assessed at baseline and at weeks 1, 2, 4, 8, and 12. The primary endpoint was the change in hemoglobin level from baseline to week 4, assessed using a one-sample t-test against a threshold of 0.5 g/dL. Secondary outcomes included longitudinal changes in hemoglobin, iron-related parameters, and quality of life from baseline to each post-treatment time point, analyzed using a linear mixed-effects model. The occurrence of adverse events was observed over 12 weeks.

**Results:**

The mean hemoglobin levels significantly increased by + 0.50 g/dL (95% confidence interval: 0.18–0.82, *P* = 0.003) from baseline to week 4, and the increase was maintained through week 12. All iron-related parameters peaked by week 2 and subsequently declined; however, serum ferritin levels remained significantly elevated at week 12. SF-36 vitality, general health and mental health scores increased significantly at 4 weeks compared with baseline. No serious adverse events were observed, although one case of arteriovenous fistula occlusion occurred.

**Conclusions:**

Single intravenous iron therapy with ferric carboxymaltose containing 500 mg of iron effectively increased hemoglobin levels without serious adverse events. Further long-term clinical trials are warranted to confirm the efficacy and safety of periodic ferric carboxymaltose therapy in patients undergoing hemodialysis.

**Trial registration:**

This study was prospectively registered with the Japan Registry of Clinical Trials on 23 May, 2023 (registration number jRCTs051230025).

**Supplementary Information:**

The online version contains supplementary material available at https://doi.org/10.1186/s12882-026-05153-z.

## Introduction

Iron-deficiency anemia is frequently observed in patients undergoing hemodialysis (HD) owing to blood loss from the dialysis circuit, reduced iron absorption, and increased iron requirements associated with the treatment of renal anemia using erythropoietin-stimulating agents (ESA) [1]. The 2015 guidelines on renal anemia in chronic kidney disease by the Japanese Society for Dialysis Therapy recommend that the iron status, assessed using serum ferritin level and transferrin saturation (TSAT), should be regularly evaluated and initiation of iron therapy when serum ferritin level is < 100 ng/mL and TSAT is < 20% in patients receiving ESA who cannot maintain the target hemoglobin (Hb) level [2].

In clinical practice, both oral and intravenous iron replacement therapies are available for patients undergoing HD. However, oral iron supplements have several disadvantages such as frequent gastrointestinal symptoms (e.g. nausea and vomiting), poor absorption rate, low compatibility with other medications, and an increased burden of medications [3]. In contrast, these adverse effects are less frequently observed in intravenous iron therapy, which has the advantage of easy administration in patients undergoing HD. Therefore, intravenous iron therapy is generally preferred for iron replacement in patients undergoing HD. The aforementioned Japanese guidelines recommend that intravenous administration of saccharated iron oxide at a dose of 40 mg once weekly or every 2 weeks for a total of 13 doses [2].

Recently, ferric carboxymaltose (FCM), a novel parenteral iron formulation, is clinically available for the treatment of iron-deficiency anemia in Japan. The FCM complex comprises a polynuclear ferric hydroxide complexed with carboxymaltose. As a stable iron complex, FCM can be administered at high doses, releases minimal free iron into the circulation [4]. In Japan, FCM infusion therapy at a weekly dose of 500 mg iron is approved by the national health insurance system for patients with iron-deficiency anemia. Therefore, iron replacement therapy using FCM in patients undergoing HD may allow for less frequent administration than conventional low-dose iron agents, which could reducetime and healthcare resource use. However, several concerns have been raised regarding the safety of higher-dose iron infusion due to the possibility of iron overload, oxidative stress, cardiovascular disease, and hypersensitivity reactions [2, 5, 6]. Therefore, widespread implementation of high-dose iron infusion therapy using FCM in Japanese patients undergoing HD requires confirmation of its safety and efficacy.

This pilot study aimed to investigate the efficacy and safety of a single-dose intravenous infusion of FCM containing 500 mg of iron in Japanese patients with iron-deficiency anemia undergoing HD, as a preliminary step towards conducting a future long-term clinical trial involving periodic intravenous iron therapy using FCM in a clinical setting.

## Materials and methods

### Study protocol

A 12-week, prospective, multicenter, single-arm, open-label study was conducted. Patients with iron-deficiency anemia undergoing maintenance HD three times a week at hemodialysis centers associated with Wakayama Medical University (Wakayama, Japan) were screened for this study. Patients aged 18 years or older with iron-deficiency anemia, as defined by a Hb < 10 g/dL and evidence of iron-deficiency (serum ferritin level < 100 ng/mL or TSAT < 20%), were eligible for this study. Patients who had received anemia treatment with an ESA or a hypoxia-inducible factor–prolyl hydroxylase (HIF-PH) inhibitor at screening were enrolled if they had not experienced any dosage changes for over 4 weeks prior to screening. The exclusion criteria during screening were as follows: patients (i) with a history of malignancy within the past 5 years, (ii) scheduled for treatment requiring hospitalization, (iii) scheduled for surgery required a blood transfusion, (iv) with pulmonary congestion on chest radiography, (v) with signs of active bleeding (e.g. gastrointestinal hemorrhage), and (vi) deemed unsuitable by the physician.

After obtaining informed consent from each patient, FCM containing 500 mg of iron was administrated intravenously (baseline: week 0) and the patients were observed for 12 weeks. The FCM was administered as a continuous intravenous infusion of FCM diluted in 100 mL of normal saline for at least 6 min. If an ESA or a HIF-PH inhibitor was used at the time of providing informed consent, the same dose of these agents was continued until 4 weeks after FCM administration. However, if the Hb level increased by more than + 1.0 g/dL over the preceding 2 weeks or exceeded 12.0 g/dL, the dose of these agents could be reduced at the physician’s discretion. After 4 weeks, ESA or HIF-PH inhibitor dose adjustments were permitted at the discretion of the physician, if deemed medically necessary. During the study period, the use of intravenous iron agents other than FCM, oral iron agents, and iron-containing medications for the treatment of hyperphosphatemia was not allowed. Patients undergoing blood transfusions or major surgeries were considered withdrawn.

Iron-related parameters including Hb, serum ferritin, TSAT, phosphate, 8-hydroxydeoxyguanosine (8-OHdG), hepcidin, and intact fibroblast growth factor 23 (FGF-23) were measured at baseline and at weeks 1, 2, 4, 8, and 12. Serum 8-OHdG (ng/mL), hepcidin (ng/mL), and intact FGF-23 (pg/mL) levels were measured using a competitive enzyme-linked immunosorbent assay (Japan Institute for the Control of Aging, Shizuoka, Japan), a latex agglutination turbidimetric immunoassay (Fujifilm Wako Pure Chemical Corporation, Osaka, Japan), and a chemiluminescent immunoassay (Canon Medical Diagnostics Corporation, Kanagawa, Japan), respectively. All parameters were measured centrally at Mediford Co., Ltd. (Tokyo, Japan).

Health-related quality of life (QOL) was evaluated using the Short Form-36 Health Survey (SF-36) version 2 [7], using the norm-based scores along with the eight domain scores (physical functioning, role physical, bodily pain, general health, vitality, social functioning, role emotional, and mental health), at baseline and at weeks 4, 8, and 12, and the EuroQol-5Dimention-5Level (EQ-5D-5 L) [8] at baseline and at weeks 1, 2, 4, 8, and 12.

The study protocol was approved by the Ethics Committee of the Wakayama Medical University (approval number: w-57). The study adhered to the Declaration of Helsinki, and all patients were fully informed and provided written informed consent. The trial was registered with the Japan Registry of Clinical Trails (registration number: jRCTs 051230025 on 23 May, 2023).

### Efficacy and safety assessments

The primary efficacy outcome of this study was the change in Hb levels from baseline (week 0) to week 4 after FCM administration. Secondary efficacy outcomes included changes in Hb level, serum ferritin, TSAT, and health-related QOL at the indicated study appointments, compared with baseline levels. Safety assessments were evaluated based on the prevalence of adverse events and the proportion of patients with serum ferritin ≥ 500 ng/mL or TSAT ≥ 50% at weeks 1, 2, 4, 8, and 12. Trajectories of serum 8-OHdG, hepcidin, intact FGF-23, and phosphate levels during the study period were also evaluated.

### Statistical analyses

Since Hb levels show minimal improvement without treatment, a threshold Hb increase of 0 g/dL was set in this study. The expected mean increase in Hb with FCM was assumed to be 0.5 g/dL, with a standard deviation of 0.90 g/dL. Using a one‑sample, one‑sided t‑test at α = 0.025 to test whether the mean Hb change at week 4 exceeds 0.50 g/dL, the required sample size to achieve 80% power was calculated as 28 patients. The target enrollment was set at 30 patients to account for possible exclusions owing to ineligibility or other reasons.

Continuous variables are summarized using means and standard deviations, and categorical variables are summarized using frequencies and proportions. The primary efficacy outcome, defined as the increase in Hb at 4 weeks after FCM administration, was evaluated using a one-sample t-test against a threshold of 0.5. Subgroups were defined as follows: sex; age, presence of diabetes mellitus, body mass index (BMI), serum iron, total iron binding capacity (TIBC), TSAT, and serum ferritin (each dichotomized at the median), and darbepoetin alfa dose (dichotomized at the median) among the 22 patients receiving darbepoetin alfa. The secondary efficacy outcomes and safety assessments from baseline to each post-treatment time point were analyzed using a linear mixed-effects model, with time points as fixed effects and subjects as random effects. Serum ferritin, TSAT, hepcidin and intact FGF-23 levels showed marked departures from normality, and log transformation was used for analysis. Statistical analyses were performed using R version 4.2.2 (R Foundation for Statistical Computing, Vienna, Austria).

## Results

The clinical characteristics of the 30 patients enrolled at the time of study registration are presented in Table 1. The mean age was 70.6 ± 12.6 years, and 56.7% were male. The mean values for Hb, ferritin and TSAT were 9.4 ± 0.4 g/dL, 85.5 ± 96.8 ng/mL and 18.5 ± 10.6%, respectively. Of the 30 participants, 96.7% received an ESA or a HIF-PH inhibitor at baseline. 18 patients received the infusion via the venous drip chamber of the extracorporeal dialysis circuit, whereas the remaining 12 patients received FCM intravenously outside the circuit. All patients were observed for 12 weeks after the treatment.

**Table 1:** Clinical characteristics of the patients at the time of study registration

Characteristics	*n* = 30
Age (years)	70.6 ± 12.6
Sex (male), n (%)	17 (56.7)
Height (cm)	160.4 ± 8.3
Body weight (kg)	58.5 ± 12.3
BMI (kg/m^2^)	22.6 ± 4.0
Hb (g/dL)	9.4 ± 0.4
Ferritin (ng/mL)	85.5 ± 96.8
TSAT (%)	18.5 ± 10.6
ESA or HIF-PH inhibitor, n (%)	29 (96.7)
ESA, n (%)	27 (93.1)
HIF-PH inhibitor, n (%)	2 (6.9)
Diabetes, n (%)	13 (43.3)
Hypertension, n (%)	27 (90.0)
Cardiovascular disease, n (%)	12 (40.0)

Continuous variables are expressed as mean ± standard deviation

BMI, body mass index; Hb, hemoglobin; TSAT, transferrin saturation; ESA, erythropoiesis-stimulating agent; HIF-PH, hypoxia-inducible factor prolyl hydroxylase

### Primary efficacy outcome

**Fig. 1:**
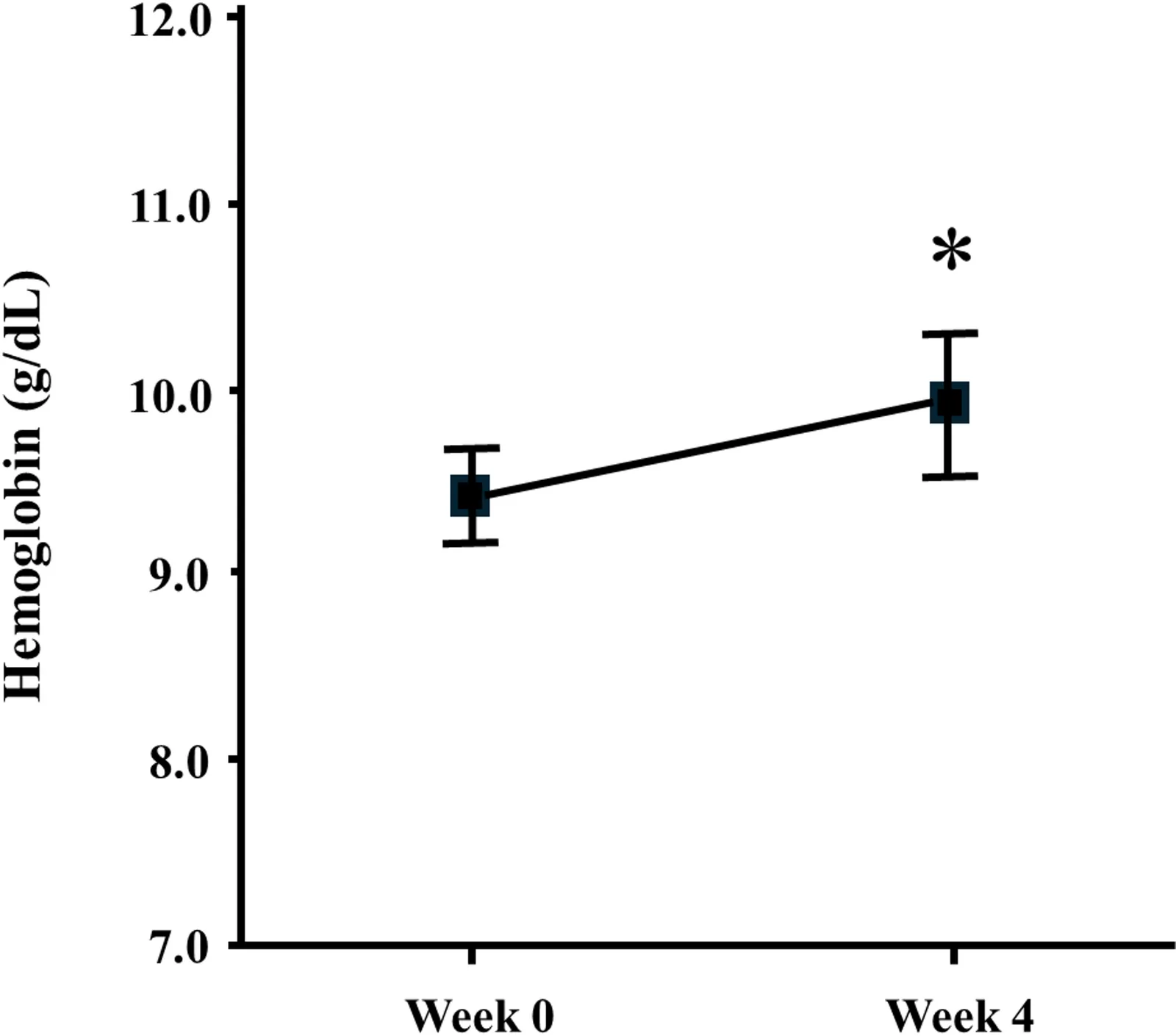
Changes in hemoglobin (Hb) levels from baseline (week 0) to week 4 in all participants. Data are expressed as mean ± standard error of the mean (SEM). * *P* = 0.003 by a one-sample t-test against a threshold of 0.5

At baseline (week 0), the mean Hb level was 9.34 ± 0.66 g/dL in 30 patients. The mean Hb value increased to 9.84 ± 1.04 g/dL at week 4 after the intravenous infusion of FCM. The primary efficacy endpoint was + 0.50 g/dL (95% CI: 0.18–0.82, *P* = 0.003), representing a significant increase compared with the baseline level (Fig. 1). When the primary efficacy endpoint was analyzed based on the pre-specified subgroups, significant increases in Hb levels at week 4 after treatment were observed in all subgroups (Table 2).

**Table 2:** Changes in Hb levels from baseline (week 0) to week 4 in pre-specified subgroups

		*n*	Hb at baseline	Hb at week 4	Changes in Hb
Subgroups					
Sex	Male	17	9.35 ± 0.73	9.94 ± 1.05	0.59 (0.18–1.00) *
	Female	13	9.32 ± 0.60	10.22 ± 0.95	0.90 (0.36–1.44) *
Age (years)	≥ 70	14	9.21 ± 0.77	9.81 ± 0.90	0.59 (0.16–1.03) *
	< 70	16	9.44 ± 0.56	10.28 ± 1.06	0.84 (0.35–1.33) *
BMI (kg/m^2^)	≥ 22.5	15	9.49 ± 0.65	10.20 ± 0.72	0.71 (0.35–1.07) *
	< 22.5	15	9.19 ± 0.67	9.92 ± 1.23	0.73 (0.16–1.30) *
Serum iron (µg/dL)	≥ 45	15	9.65 ± 0.56	10.47 ± 1.04	0.81 (0.29–1.34) *
	< 45	15	9.02 ± 0.62	9.65 ± 0.81	0.63 (0.22–1.05) *
TIBC (µg/dL)	≥ 250	16	9.21 ± 0.68	10.15 ± 1.21	0.94 (0.46–1.42) *
	< 250	14	9.48 ± 0.63	9.96 ± 0.73	0.48 (0.06–0.90) *
TSAT (%)	≥ 18	15	9.55 ± 0.60	10.25 ± 0.78	0.70 (0.35–1.05) *
	< 18	15	9.12 ± 0.67	9.87 ± 1.18	0.75 (0.17–1.32) *
Serum ferritin (ng/mL)	≥ 60	14	9.62 ± 0.62	10.16 ± 0.82	0.54 (0.17–0.90) *
	< 60	16	9.09 ± 0.62	9.97 ± 1.16	0.89 (0.36–1.41) *
Diabetes	Yes	13	9.18 ± 0.74	9.71 ± 1.02	0.53 (0.19–0.87) *
	No	17	9.46 ± 0.59	10.33 ± 0.93	0.87 (0.36–1.38) *
Darbepoetin alfa (µg/week)	≥ 18	7	8.97 ± 0.71	10.33 ± 1.52	1.36 (0.33–2.39) *
	< 18	15	9.56 ± 0.50	10.06 ± 0.86	0.50 (0.07–0.93) *

Data (Hb level, g/dL) are expressed as mean ± standard deviation or mean (95% confidence interval)

* indicates a significant difference (*P* < 0.05) in changes in Hb levels from baseline to week 4

BMI, body mass index; Hb, hemoglobin; TIBC, total iron-binding capacity; TSAT, transferrin saturation

### Secondary efficacy outcomes

The mean Hb level continued to increase beyond week 4, rising by + 0.70 g/dL (95% CI: 0.39–1.01, *P* < 0.001) at week 12 compared with the baseline level (Table 3). After dose adjustments of the ESA or HIF-PH inhibitor were permitted to maintain the target Hb levels, the dosage of ESA was decreased in one patient and increased in four patients. In contrast, serum ferritin levels increased significantly at week 1 and declined thereafter, although they remained significantly above the baseline level until week 12 (Table 3). TSAT also increased significantly at week 1 compared with the baseline level, then declined gradually, and no significant differences were observed at week 12 (Table 3). In the SF-36, the scores for vitality and mental health increased significantly at weeks 4, 8, and 12 compared with the baseline level (Table 4). The role-physical function scores significant decreased only at week 8. General health scores increased significantly only at week 4. In contrast, no changes in EQ-5D-5 L index scores were observed during the study period (Supplementary Figure S1).

**Table 3:** Trajectories of Hb and iron-related parameters during the study period

	Baseline (week 0)	week 1	week 2	week 4	week 8	week 12
Hb						
Mean (95% CI)	9.34 (9.09–9.58)	9.48 (9.18–9.78)	9.54 (9.26–9.82)	9.84 (9.45–10.23)	10.06 (9.69–10.43)	10.04 (9.63–10.44)
* P* value		0.294	0.136	< 0.001	< 0.001	< 0.001
Ferritin						
Median (IQR)	67 (33–153)	520 (383–814)	372 (232–585)	241 (105–350)	136 (48–239)	107 (29–197)
* P* value *		< 0.001	< 0.001	< 0.001	< 0.001	0.004
TSAT						
Median (IQR)	18.5 (12.3–27.0)	27.0 (21.0–29.8)	25.5 (19.8–36.0)	24.0 (18.8–27.3)	18.5 (16.0–29.8)	21.5 (13.8–29.0)
* P* value *		< 0.001	< 0.001	0.001	0.030	0.219
8-OHdG						
Mean (95% CI)	0.29 (0.25–0.33)	0.36 (0.32–0.40)	0.33 (0.29–0.38)	0.32 (0.28–0.36)	0.31 (0.27–0.35)	0.29 (0.25–0.33)
* P* value		< 0.001	0.010	0.084	0.227	0.86
Hepcidin						
Median (IQR)	10.7 (3.0–53.6)	51.5 (17.3–92.9)	39.1 (18.6–78.7)	34.3 (9.4–85.9)	36.9 (3.0–66.6)	27.5 (3.0–71.9)
* P* value *		< 0.001	< 0.001	< 0.001	0.007	0.037
intact FGF23						
Median (IQR)	693 (274–3638)	1360 (526–3755)	987 (262–2698)	711 (263–2698)	478 (227–3690)	654 (210–2593)
* P* value *		< 0.001	0.054	0.359	0.197	0.432
Phosphate						
Mean (95% CI)	5.06 (4.59–5.53)	4.77 (4.32–5.23)	5.08 (4.58–5.58)	5.16 (4.60–5.72)	4.90 (4.39–5.41)	5.06 (4.45–5.68)
* P* value		0.106	0.910	0.573	0.367	0.985

Data are expressed as mean (95% confidence interval) or median (interquartile range)

*P* values are provided using a linear mixed-effects model

* *P* value is the result of an analysis using the log-transformed values

CI, confidence interval; FGF-23, fibroblast growth factor 23; Hb, hemoglobin; IQR, interquartile range; 8-OHdG, 8-hydroxydeoxyguanosine; TSAT, transferrin saturation

**Table 4:** Trajectories in norm-based scores of the eight domains of SF-36 during the study period

	baseline	week 4	week 8	week 12
Physical functioning	32.83 ± 6.19	32.37 ± 6.11	30.64 ± 6.12	31.82 ± 6.08
Role (physical)				
Bodily pain	43.08 ± 5.07	43.72 ± 4.78	43.88 ± 5.28	43.62 ± 5.23
General health	45.57 ± 4.25	47.97 ± 4.30*	45.96 ± 4.16	45.93 ± 4.02
Vitality	46.09 ± 4.20	49.13 ± 4.58*	49.84 ± 4.17*	49.03 ± 4.28*
Social functioning	44.77 ± 5.11	45.14 ± 5.24	46.63 ± 4.59	47.58 ± 4.69
Role (emotional)	46.53 ± 5.08	44.54 ± 5.42	43.55 ± 5.34	43.56 ± 5.58
Mental health	49.40 ± 4.10	52.27 ± 3.78*	52.34 ± 3.81*	52.43 ± 4.31*

Data (norm-based scores) are expressed as mean ± standard deviation

* *P* < 0.05, vs. baseline using a linear mixed-effects model

### Safety assessments

During the study period, no serious adverse events, such as infections, cardiovascular diseases, surgical procedures, hospitalizations, or deaths were observed. Although one case of an arteriovenous fistula occlusion occurred, it was concluded that there was no causal relationship with the investigational treatment. Sixteen patients (53.3%) had serum ferritin levels of over 500 ng/mL at week 1 after treatment (Supplementary Table S1). After week 8, all patients had serum ferritin levels below 500 ng/mL, except for one patient in whom ferritin level was already above 500 ng/mL even prior to treatment and remained at these levels throughout the study. This patient was included in the study because of a baseline TSAT < 20%. Also, two patients (6.7%) had TSAT > 50% at week 1 (Supplementary Table S1). After week 2, TSAT fell below 50% in all patients, except one patient who had already presented a TSAT > 50% prior to treatment. This patient was included in the study because of a baseline serum ferritin level of < 100 ng/mL. Serum levels of 8-OHdG, hepcidin, and intact FGF-23 peaked at week 1, after which they declined and returned to baseline levels by week 12 (Table 3); however, hepcidin levels were still statistically higher at week 12 than that at baseline. Serum phosphate levels did not increase or decrease at any time point during the study period (Table 3).

## Discussion

This pilot study, comprising 30 Japanese patients with iron-deficiency anemia undergoing maintenance HD, revealed that a single-dose administration of FCM containing 500 mg of iron significantly increased Hb levels by an average of + 0.50 g/dL at week 4. This increase was sustained throughout the observation period, with an additional increase of + 0.70 g/dL by week 12. This trajectory of Hb levels in response to FCM injection was consistent with a previous analysis [7]. In contrast, serum ferritin and TSAT levels peaked within 2 weeks of administration, declining thereafter, and returning to baseline levels by week 12. Similarly, the serum concentrations of 8-OHdG, hepcidin, and intact FGF-23 peaked at week 1, after which they declined. Furthermore, no serious adverse events occurred during the study period, although one adverse event (arteriovenous fistula occlusion) was observed. The rate of arteriovenous fistula occlusion observed in this study was not considered to be higher than that generally observed in Japanese patients undergoing HD. Therefore, these observations suggest that a single intravenous therapy using FCM containing 500 mg iron is well-tolerated and can effectively and safely replenish iron in Japanese patients undergoing HD. However, the small sample size, short observation period, and a single administrationof FCM do not allow to reach conclusive safety conclusions.

Several observational studies have raised safety concerns that high-dose intravenous iron therapies such as monthly doses exceeding 300 mg are associated with a higher risk of infection, cardiovascular mortality, all-cause death, and atherothrombosis [9, 10]. However, a meta-analysis that investigated the safety of higher-dose versus lower-dose intravenous iron, oral iron, or no iron supplementation in patients undergoing dialysis reported that higher-dose intravenous iron was not associated with high risk of mortality, infection, cardiovascular events, or hospitalizations [11], although the strength of this finding is limited by the small number of participants and events in the randomized controlled trials and statistical heterogeneity in observational studies. A recent randomized control trial (PIVOTAL: the Proactive IV Iron Therapy in Hemodialysis Patients) demonstrated that the use of a proactive high-dose intravenous iron regimen was associated with a lower risk of death or major adverse cardiovascular events than that observed with the use of a reactive low-dose intravenous iron regimen [12]. The PIVOTAL trial also showed that the high-dose group, which received a monthly dose of 400 mg, had a significantly lower dose of ESA and lower incidence of blood transfusion, whereas the incidence of infection and hospitalization for any cause did not differ significantly between the two groups. These results suggest that intravenous iron therapy at doses higher than those currently used in many regions worldwide, including Japan, has cardiovascular benefits in patients undergoing HD.

Serum ferritin levels and TSAT are clinically used to evaluate iron status and avoid iron overload. High serum ferritin levels and TSAT have been discussed in relation to increased mortality. However, observational studies have revealed conflicting results regarding the impact of higher serum ferritin levels on the mortality of patients undergoing HD [13–17]. Whether transient elevations, but not sustained increases, in serum ferritin levels and TSAT contribute to mortality and morbidity risks remained unclear. In our study, a single-dose administration of FCM rapidly elevated serum ferritin levels and TSAT by week 1. At week 1, serum ferritin levels exceeded 500 ng/mL in half of the patients, and TSAT exceeded 50% in two patients. However, these levels gradually declined after week 4 in most patients, unlike Hb levels. These trajectories of serum ferritin levels and TSAT after the high-dose intravenous iron infusion are compatible with the observations of previous studies [18, 19]. Bielesz et al. reported an open-label, randomized controlled study over 40 weeks that compared FCM therapy containing 500 mg of iron every 10 weeks with biweekly 100 mg iron-sucrose therapy in patients undergoing HD [20]. Although non-inferiority in Hb change was not demonstrated, the 10-week FCM regimen did not cause progressive accumulation of serum ferritin or TSAT over the study period. Furthermore, the overall number of adverse events was similar, and there was a trend toward a higher incidence of infections in the biweekly iron-sucrose regimen than that in the 10-week FCM regimen. Taken together, these observations suggest that the administration of FCM at relatively long intervals is unlikely to result in prolonged iron overload and cause adverse events in patients undergoing HD.

Average serum ferritin levels differ among regions worldwide. The International Dialysis Outcomes and Practice Patterns Study (DOPPS) showed median ferritin levels of 718 ng/mL in the United States and 405 ng/mL in Europe, whereas it was 83 ng/mL in Japan [21]. However, even among Japanese patients registered in this DOPPS, at least the adjusted risk in the group categorized with the highest levels of serum ferritin (average serum ferritin of 415 ± 311 ng/mL) was not significantly higher than that in the reference group (83 ng/mL). Recently, the average serum ferritin level of patients undergoing HD in the United States has increased to approximately 800 ng/mL, partly due to the increased use of intravenous iron supplementation to compensate for the reduced ESA dosage [22]. Thus, the results obtained from other regions cannot be applied to Japanese patients. Therefore, we conducted this pilot study using a single-dose administration of FCM containing 500 mg iron to confirm the efficacy and safety of high-dose intravenous iron therapy in Japanese patients undergoing HD before conducting a long-term study with periodic FCM therapy.

Higher levels of serum 8-OHdG are associated with all-cause mortality in patients undergoing HD [23, 24]. Maruyama et al. reported that repeated intravenous administration of ferric saccharate caused prolonged elevation of serum 8-OHdG levels for up to 10 weeks [25]. This indicates that frequent intravenous iron infusions cause accumulation of serum 8-OHdG, resulting in prolonged exposure to oxidative stress and an increased risk of mortality. Our study showed that significant elevations in 8-OHdG levels were observed at week 1 following FCM infusion, after which they declined. This trajectory suggests that FCM therapy with a long-term interval may attenuate prolonged and accumulated exposure to oxidative stress compared with weekly ferric saccharate administration. However, the optimal administration interval for periodic high-dose intravenous iron therapy to safely maintain Hb levels while avoiding oxidative stress accumulation remains unclear. Further studies are required to confirm whether periodic high-dose intravenous iron therapy using FCM induces the accumulation of these iron-related parameters.

Iron-induced hypophosphatemia has been reported in the literature [26]. This adverse event is not a class-effect observed across all intravenous iron preparations but is linked to specific intravenous iron formulations, particularly FCM [27, 28]. The mechanism of the FCM-induced hypophosphatemia is hypothesized as FCM inhibits the proteolytic cleavage of FGF-23, resulting in increased urinary phosphate excretion being mediated by increased FGF-23, which subsequently causes hypophosphatemia following FCM infusion. Interestingly, a study reported that the incidence of FCM-induced hypophosphatemia negatively correlated with estimated glomerular filtration rate [29], indicating that patients with impaired renal function were at a lower risk of developing FCM-induced hypophosphatemia than those with normal renal function. Our study also demonstrated that despite a temporary increase in intact FGF-23 levels, serum phosphate levels remained stable throughout the study period in patients undergoing HD. Therefore, patients with severely impaired renal function, such as those with end-stage renal disease, are considered to have FCM-induced hypophosphatemia less frequently.

The present study also assessed patient-reported outcomes using the SF-36 and EQ-5D-5 L to investigate the impact of high-dose iron infusion on patients’ QOL. Among the SF-36 domains, vitality and mental health scores improved significantly at weeks 4, 8, and 12 compared with baseline. Similarly, a study on Japanese patients undergoing HD showed that anemia correction with darbepoetin alfa was associated with improvements in SF-36 vitality and mental health scores [30]. Long-term observation is needed to confirm these effects of high-dose iron therapy on the QOL in patients undergoing HD.

This study has some limitations that must be addressed. First, the sample size was small (30 patients), and the observation period was short (12 weeks). Although enrolling 30 patients provided adequate power to detect the pre-specified primary endpoint, the sample size was insufficient to reliably assess adverse events. Second, only a single dose of FCM was administered. Therefore, it remains unclear whether repeated iron therapy using FCM leads to an accumulation of iron-related indices, such as serum ferritin and TSAT, which cause adverse effects. In addition, the interval between subsequent doses of periodic iron therapy using FCM necessary to avoid iron overload in Japanese patients undergoing HD is unclear. Currently, no protocol has been established for FCM therapy in patients undergoing HD.

## Conclusions

We confirmed the efficacy and safety of a single-dose administration of FCM containing 500 mg iron in Japanese patients undergoing HD. FCM proved effective in producing clinically meaningful increases in Hb levels without serious adverse events. These findings suggest that FCM is a reliable option for managing iron repletion and anemia in Japanese patients undergoing HD. Further long-term studies are warranted to confirm the efficacy and safety of periodic iron therapy using FCM in Japanese patients undergoing HD.

## Supplementary Information

Below is the link to the electronic supplementary material.

Supplementary Material 1

## Data Availability

All clinical data supporting the findings of this study are available on reasonable request to the corresponding author and approval by the Wakayama Medical University Ethics Committee.

## Abbreviations

DOPPS: International Dialysis Outcomes and Practice Patterns Study
EQ-5D-5L: EuroQol 5-Dimension 5-Level
ESA: Erythropoietin-stimulating agents
FCM: Ferric carboxymaltose
FGF-23: Fibroblast growth factor 23
Hb: Hemoglobin
HD: Hemodialysis
HIF-PH: Hypoxia-inducible factor–prolyl hydroxylase
PIVOTAL: Proactive IV Iron Therapy in Hemodialysis Patients
QOL: Quality of life
SF-36: Short Form-36 Health Survey
TSAT: Transferrin saturation
8-OHdG: 8-hydroxydeoxyguanosine
