# Supplementary material for: Efficacy and safety of single high-dose intravenous ferric carboxymaltose in patients undergoing hemodialysis: a multicenter, single-arm, open-label, clinical trial
Source: BMC Nephrol. 2026 Jun 29;27:543. doi: 10.1186/s12882-026-05153-z (PMC13584270; doi:10.1186/s12882-026-05153-z)
Supplement: Supplementary file 1 [file 12882_2026_5153_MOESM1_ESM.docx]

**Supplementary table S1. The number of patients who exceeded 500 ng/mL of serum ferritin or 50% of TSAT at each observation point.**

|  | baseline | week 1 | week 2 | week 4 | week 8 | week 12 |
| --- | --- | --- | --- | --- | --- | --- |
| Total (n) | 30 | 30 | 30 | 30 | 30 | 30 |
| Patients with ferritin over 500 ng/mL (n) | 1 | 16 | 9 | 4 | 1 | 1 |
| Patients with TSAT over 25% (n) | 1 | 2 | 1 | 1 | 1 | 1 |

TSAT, transferrin saturation.

**Supplementary Figure S1.**

**Supplementary Figure S1. Trajectories in EQ-5D-5L visual analogue scale during study period.**

Data are expressed as mean ± standard error of the mean (SEM).

No significant changes in EQ-5D-5L index scores were observed over the study compared to baseline (week 0) using a linear mixed-effects model.
